# microRNA-29c inhibits cell proliferation by targeting NASP in human gastric cancer

**DOI:** 10.1186/s12885-017-3096-9

**Published:** 2017-02-07

**Authors:** Beiqin Yu, Xuehua Chen, Jianfang Li, Qinlong Gu, Zhenggang Zhu, Chen Li, Liping Su, Bingya Liu

**Affiliations:** Shanghai Key Laboratory of Gastric Neoplasms, Department of Surgery, Shanghai Institute of Digestive Surgery, Ruijin Hospital, Shanghai Jiao Tong University School of Medicine, No.197 Ruijin Er Road, Shanghai, 200025 People’s Republic of China

**Keywords:** miR-29c, NASP, Gastric cancer, Proliferation

## Abstract

**Background:**

Gastric cancer is one of the most common malignancies worldwide. Recent studies have shown that microRNAs play crucial roles in regulating cellular proliferation process in gastric cancer. MicroRNA-29c (miR-29c) acts as a tumor suppressor in different kinds of tumors.

**Methods:**

Quantitative PCR was performed to evaluate miR-29c expression level in 67 patient gastric cancer tissues and 9 gastric cancer cell lines. The effects of miR-29c were explored by proliferation assay, soft agar colony formation assay, apoptosis and cell cycle analysis using flow cytometry. The target gene was predicted by bioinformatic algorithms and validated by dual luciferase reporter assay and Western blot analysis.

**Results:**

In this study, we demonstrate that miR-29c is down-regulated in gastric cancer tissues and cell lines. We indicate that overexpression of miR-29c inhibits cell proliferation, promotes apoptosis and arrests cell cycle at G1/G0 phase. We additionally show that miR-29c exerts these effects by targeting Nuclear autoantigenic sperm protein (NASP). Moreover, depletion of NASP can elite the phenotypes caused by miR-29c.

**Conclusions:**

These data suggest that miR-29c inhibits proliferation in gastric cancer and could potentially serve as an early biomarker and a novel therapy target.

**Electronic supplementary material:**

The online version of this article (doi:10.1186/s12885-017-3096-9) contains supplementary material, which is available to authorized users.

## Background

Gastric cancer is the fifth most common malignancy and the third leading cause of cancer-related deaths, according to the GLOBOCAN series of the International Agency for Research on Cancer [1]. Surgery is the only curative treatment, however, many patients have inoperable disease at diagnosis or have recurrent disease after resection [2]. Therefore, it is crucial to elucidate the molecular mechanisms underlying the development of gastric cancer and to look for new molecular markers and therapeutic targets.

MicroRNAs (miRNAs) are a class of small, non-coding RNAs about 18–25 nucleotides in length. MiRNAs mainly function to negatively regulate gene expression by promoting mRNA degradation or inhibiting mRNA translation through interacting with perfect or imperfect complementary sequences between the miRNA seed and the 3′untranslated regions (3′UTR) of its target genes [3]. MiR-29c belongs to the miR-29 family, which is composed of four species with identical seed sequences, namely miR-29a, miR-29b-1, miR-29b-2 and miR-29c [4]. MiR-29c plays the role as tumor suppressor in several kinds of tumors. MiR-29c was shown to inhibit cell growth, cell migration and invasion in pancreatic cancer by targeting ITGB1 [5]. In bladder cancer, miR-29c overexpression inhibited cell growth, suppressed cell migration and resulted in an accumulation of cells in the G1 phase during the cell cycle through the target gene CDK6 [6]. MiR-29c was displayed to mediate the epithelial to mesenchymal transition (EMT) and negatively regulated Wnt/β-catenin signaling pathway via PTP4A and GNA13 in human colorectal carcinoma [7]. MiR-29c down-regulation by CpG dinucleotide methylation of the promoter has been participated in cell invasion and increased sensitive to chemotherapy in basal-like breast tumors [8]. Further studies on liver carcinoma that focused on the suppressive role of ionizing radiation-responsive miR-29c in the development of the disease via targeting WIP1 [9]. In lung cancer, miR-29c was shown to suppress cell adhesion and metastasis by targeting integrin β1 and MMP2 [10].

In the present study, we qualified the expression of miR-29c in gastric cancer tissues and evaluated its role in cell proliferation and induction of cell apoptosis. We found that miR-29c has a general decrease in gastric cancer tissues compared with the matched normal tissues. Overexpression of miR-29c reduced cell proliferation by promoting apoptosis and inducing cell cycle G1/G0 arrest in vitro, and inhibited the ability of tumorigenesis in gastric cancer cells in vivo. Furthermore, we demonstrated that miR-29c can decrease NASP expression and the effects observed following miR-29c overexpression are partially due to NASP depletion. Thus, all of these results suggest that miR-29c is a potential marker for diagnose and therapeutic target for treatment in gastric cancer.

## Methods

### Human samples

Sixty-seven pairs of tumor tissues and paired adjacent normal tissues were collected from patients with gastric cancer who underwent surgery at the Department of Surgery, Ruijin Hospital, Shanghai Jiao Tong University School of Medicine. All samples were diagnosed by pathological examination, clinicopathological data were reviewed and TNM staging classification was ranked base on criteria of American Joint Committee on Cancer (AJCC, 6^th^ edition).

### Cell lines

The human gastric cancer cell lines SNU-1 (ATCC No. CRL-5971), SNU-16 (ATCC No. CRL-5974), NCI-N87 (ATCC No. CRL-5822), AGS (ATCC No. CRL-1739) and KATOIII (ATCC No. HTB-103) were got from the American Type Culture Collection, MKN-45 (JCRB No. 0254) and MKN-28 (JCRB No. 0253) were obtained from the Japanese Cancer Research Resources Bank, and the others (BGC-823: CBP60477, SGC-7901: CBP60500) were obtained from Shanghai Institute for Biological Sciences, Chinese Academy of Science. The immortalized normal gastric mucosal epithelial cell line (GES-1) and the human embryonic kidney cell line 293 T (HEK 293 T) were preserved in our institute. Gastric cancer cell lines were cultured in RPMI-1640, while HEK 293 T cells were cultured in DEME, supplemented with 10% heat-inactivated fetal calf serum with 100 U/ml penicillin and 100 μg/ml streptomycin at 37 °C in a humidified atmosphere of 5% CO_2_. Exponentially growing cells were used for experiments.

### RNA extraction and quantitative PCR (qPCR)

Total RNA isolation from homogenized tissue samples and cell lines was performed using Trizol reagent (Invitrogen, Carlsbad, CA, USA) and reverse transcribed into cDNA using the miScript II RT Kit (Qiagen, Venlo, Limburg, Netherlands). MiR-29c qPCR was assayed by All-in-One qPCR Mix Kit (GeneCopoeia, Rockville, MD, USA) with specific primer on ABI 7900 system. Expression of miR-29c was normalized to U6 small nuclear RNA and analyzed by the 2^-ΔΔCt^ method. NASP mRNA expression level was measured by SYBR Green real time PCR (Applied Biosystems, Foster City, CA, USA) following the manufacturer’s instructions. GAPDH was used as an internal control. Following primers were used: NASP sense 5′- GCGTCCCAAATTGCCTGTTT -3′ antisense 5′- GCTTCACTATCCACATCCAGA-3′; GAPDH sense 5′-GGACCTGACCTGCCGTCTAG-3′ antisense 5′-GTAGCCCAGGATGCCCTTGA-3′.

### Transient transfection

Oligonucleotides hsa-miR-29c mimics (miR-29c), miR-control and siRNAs for NASP were purchased from GenePharma (Shanghai, China). Oligonucleotides and siRNAs were transfected into cells by carring out with Lipofectamine 2000 (Invitrogen) at a final concentration of 100 nM. The transfection efficiency was monitored by qPCR or Western blot.

### Cell proliferation assay

Cell proliferation was accessed by colorimetric water-soluble tetrazolium salt (WST) method using the Cell Counting Kit-8 (Dojindo, Kumamoto, Japan) according to the manufacturer’s instructions. After 24 h, cells transfected with miR-29c mimics or NASP siRNA were seeded into 96-well plates (2 × 10^3^ cells/well), and the proliferation was monitored everyday for 5 days. The number of viable cells was determined by measurement of the absorbance at 450 and 600 nm using a Safire 2 microplate reader (TECAN, Switzerland).

### Soft agar colony formation assay

Cells transfected with miR-29c mimics were resuspended with 0.3% soft agar in RPMI-1640 containing 10% FBS, then layered onto 0.6% solidified agar in RPMI-1640 containing 10% FBS in 6-well plates (1 × 10^3^ cells/well) at 24 h post-transfection. These plates were incubated at 37 °C for 2 weeks. Colonies containing 50 cells or more were counted.

### Apoptosis analysis

Cells transfected with miRNA or siRNA were harvested at 48 h after transfection cells and stained with Annexin V-FITC Apoptosis Detection Kit I (BD Pharmingen, CA, USA). Apoptotic cells were assessed in triplicates and repeated three times independently by flow cytometry (FACS Calibur, Becton Dickinson, NJ, USA).

### Cell cycle analysis

At 48 h post-transfection with miRNA or siRNA, cells were fixed overnight using 70% ethanol at 4 °C, washed two times in cold phosphate-buffered saline, and incubated with 100 μg/ml RNase A and 50 μg/ml propidium iodide for 1 h at 37 °C. Analysis was performed on a FACS Calibur flow cytometry by measurement of the percentage of cells in various phases of the cell cycle.

### Construction of plasmids and luciferase activity assay

Wild type NASP-3′ UTR or mutant NASP-3′ UTR containing the putative miR-29c binding sites were synthesized by Sangon, Shanghai, China. After digestion by MluI and SpeI, wild type and mutant NASP-3′ UTR were cloned into the MluI and SpeI precut pMIR-Report luciferase vector. In HEK 293 T cells pre-seeded 24-well, 100 ng pMIR/NASP-WT or MUT, together with 2 ng pRL-TK vector containing *Renilla* luciferase and 100 nM miR-29c mimics or miR-control were cotransfected by Lipofectamine 2000 (Invitrogen). After 48 h, relative luciferase activity was measured by dual-luciferase assay (Promega, Madison, WI, USA) according to the manufacturer’s instruction.

### Western blot analysis

Cells in culture were lysed using M-PER reagents (Pierce, Rockford, IL, USA) in the presence of Cocktail protease inhibitor (Pierce). The concentration was measured by a BCA Protein Assay Kit (Pierce). Fifty micrograms protein samples were resolved with 5× Lane Marker Reducing Sample Buffer (Pierce), electrophoresed in 10% SDS-PAGE and transferred onto PVDF membranes (Bio-Rad Laboratories, CA, USA). Labeled bands were detected using the ECL chemiluminescent kit (Pierce). Rabbit polyclonal anti-NASP (1:1000, Abcam, Cambridge, UK) and mouse monoclonal anti-GAPDH (1:10000, Kangchen, Shanghai, China) were used.

### Retroviral transfection for stable cell lines

A genomic region including the primary transcript of miR-29c was cloned into the EcoRI-Xhol modified pMSCV-GW-RfA-PGK-EGFP retroviral vector, no insert vector as a control. HEK 293 T cells (1 × 10^6^ cells/well) were seeded in 6-well plates 24 h prior to transfection, 2 μg of retroviral construct containing either miR-29c or miR-control, 2 μg of gag/pol and 2 μg of VSVG were cotransfected into HEK 293 T cells using 18 μl FuGENE6 HD (Roche, Indianapolis, IN, USA) in each well. At 48 and 72 h post-transfection, viruses were harvested and spin infected at 1500 rpm for 30 min at room temperaturewith 8 μg/ml of polybrene. GFP positive cells were sorted by flow cytometry and named RV-miR-29c and RV-miR-control, respectively.

### Tumor xenograft model

SGC-7901 cells (100 μl, 1 × 10^6^ cells) infected with RV-miR-29c or RV-miR-control were injected into the right flank region of 4-week-old male nude mice (Institute of Zoology, Chinese Academy of Sciences, Shanghai, China). Each group had five mice. Tumor volume was measured with caliper and calculated using the following formula: volume = (length × width^2^)/2. Mice were euthanized 4 weeks after injection and tumor nodules were removed and weighted. After tumor excision, the tumor nodules were fixed in 10% buffered formalin for further analysis. Animal study and experimental protocol was approved by the Institutional Animal Care and Use Committee of the Shanghai Jiao Tong University.

### Immunohistochemistry (IHC)

Blocks of formalin-fixed, paraffin-embedded mouse subcutaneous tumors were used. Tissue sections (5 μm) were deparaffinized with xylene, rehydrated in ethanol, antigen retrieval was performed by boiling in 10 mM citrate buffer (pH 6.0) for 30 min. After inhibition endogenous peroxidase activity with 0.3% H_2_O_2_ for 10 min, sections were blocked in 2% serum in PBS for 30 min, incubated with Ki-67 (dilution 1:50, Dako, Carpinteria, CA, USA) or NASP (dilution 1:100) at 4 °C overnight, followed by secondary antibody incubation and visualized with Envision System (Dako). Sections were counterstained with hematoxylin.

### Statistics

Experimental data were expressed as the mean ± SD. Pearson *χ* ^2^ test was applied to examine the relationship between the miR-29c expression level and clinicopathologic parameters, unpaired t test was used to analyzed the differences between two groups. All statistical analyses were performed using the SPSS 15.0 software, and a *P* value less than 0.05 was considered statistically significant.

## Results

### MiR-29c is down-regulated in gastric cancer tissues and cell lines

**Fig. 1:**
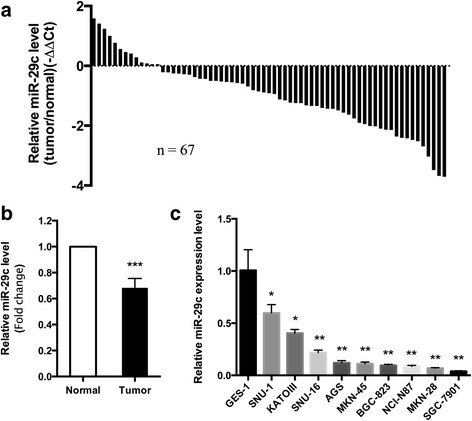
Expression of miR-29c was down-regulated in gastric cancer tissues and cell lines. **a** miR-29c expression in 67 pairs of gastric cancer tissues compared to matched adjacent normal tissues. miRNA expression of each sample was normalized to U6 expression. Normalized miRNA tumor expression was compared to normalized normal sample. **b** Expression of miR-29c in 67 pairs of gastric cancer and normal tissues. **c** miR-29c expression in GES-1 and nine gastric cancer cell lines (SNU-1, SNU-16, AGS, MKN-45, MKN-28, BGC-823, NCI-N87, KATOIII and SGC-7901). Data shown are the mean ± SD of three independent experiments. * *P* < 0.05, ** *P* < 0.01, *** *P* < 0.001

To evaluate the significance of miR-29c in gastric cancer, we first detected miR-29c expression level in 67 pairs of gastric cancer tissues and adjacent normal tissues by qPCR. As shown in Fig. 1a and b, miR-29c expression level was significantly down-regulated in gastric cancer tissues compared with matched normal tissues (*P* < 0.001). The expression levels of miR-29c in nine gastric cancer cell lines and one immortalized normal gastric mucosal epithelial cell line GES-1 were also assessed (Fig. 1c). All of the gastric cancer cell lines have a significant decrease in the levels of miR-29c compared with GES-1. Significantly, SGC-7901 expressed the lowest level of miR-29c among these cell lines. Together, these data indicate that miR-29c was prominently down-regulated in gastric cancer.

Moreover, we analyzed the relationship between the expression level of miR-29c and the clinicopathological parameters in the 67 cases. Among these samples, 80.6% (54/67) of tumor tissues showed down-regulation of miR-29c in comparison to matched normal tissues (relative expression ratio < 1.0). Furthermore, 47.8% (32/67) of tumor tissues displayed more significant down-regulation of miR-29c (relative expression ratio < 0.5). On the basis of relative expression ratio < 0.5, the 67 gastric cancer tissues were classified into two groups: low-miR-29c group (*n* = 32) and high-miR-29c group (*n* = 35). Unfortunately, miR-29c expression level did not show any correlation with gender, age, location, Borrmann classification, differentiation, local invasion, lymph node metastasis or TNM stage (Additional file 1: Table S1).

### miR-29c inhibits gastric cancer cell proliferation in vitro

**Fig. 2:**
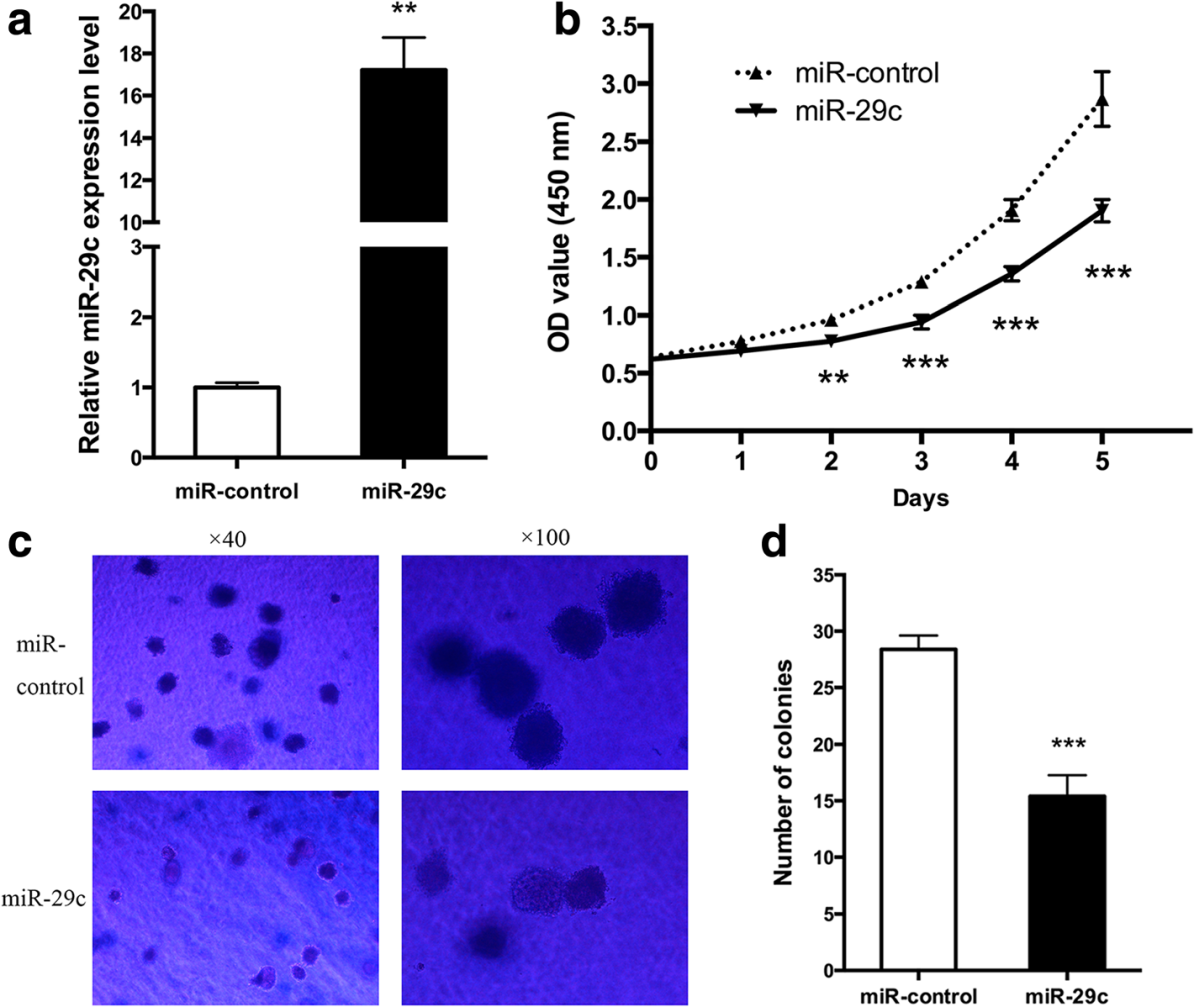
miR-29c inhibited gastric cancer cell proliferation. **a** miR-29c expression level in SGC-7901 transfected with miR-control or miR-29c detected by qPCR. **b** CCK-8 assay was performed to monitor the proliferation of SGC-7901 treated with miR-control or miR-29c. **c**, **d** Cell proliferation was measured by colony formation assay. Data are represented as the mean ± SD from three independent experiments. ** *P* < 0.01, *** *P* < 0.001

Based upon the analysis of the expression level of miR-29c in gastric cancer tissues and cell lines, we hypothesized that miR-29c re-expression might lead to an inhibition of cell growth. SGC-7901 was chosen for subsequent functional studies because of its lowest expression level of miR-29c. To test if miR-29c overexpression decreases cell viability, SGC-7901cells were transfected with 100 nM miR-29c mimics, and miR-29c was elevated by 17.2 ± 2.19 fold compared to miR-control (*P* = 0.009, Fig. 2a). The efficiency of transfection was also monitored by using a Cy3-labeled pre-miR negative control (Additional file 2: Figure S1A). As hypothesized, we found that miR-29c overexpression leads to cell growth inhibition through CCK-8 cell proliferation assay (Fig. 2b). Further study of cell viability using colony formation assay also exhibited an obvious attenuation of cell growth in SGC-7901 cells transfected with miR-29c mimics (28.4 ± 2.70 vs. 15.4 ± 4.22, *P* < 0.001, Fig. 2c and d). Taken together, these results suggest that miR-29c exerts a growth inhibitory effect in gastric cancer cells.

### miR-29c promotes gastric cancer cell apoptosis and induces cell cycle arrest in G1/G0 phase

**Fig. 3:**
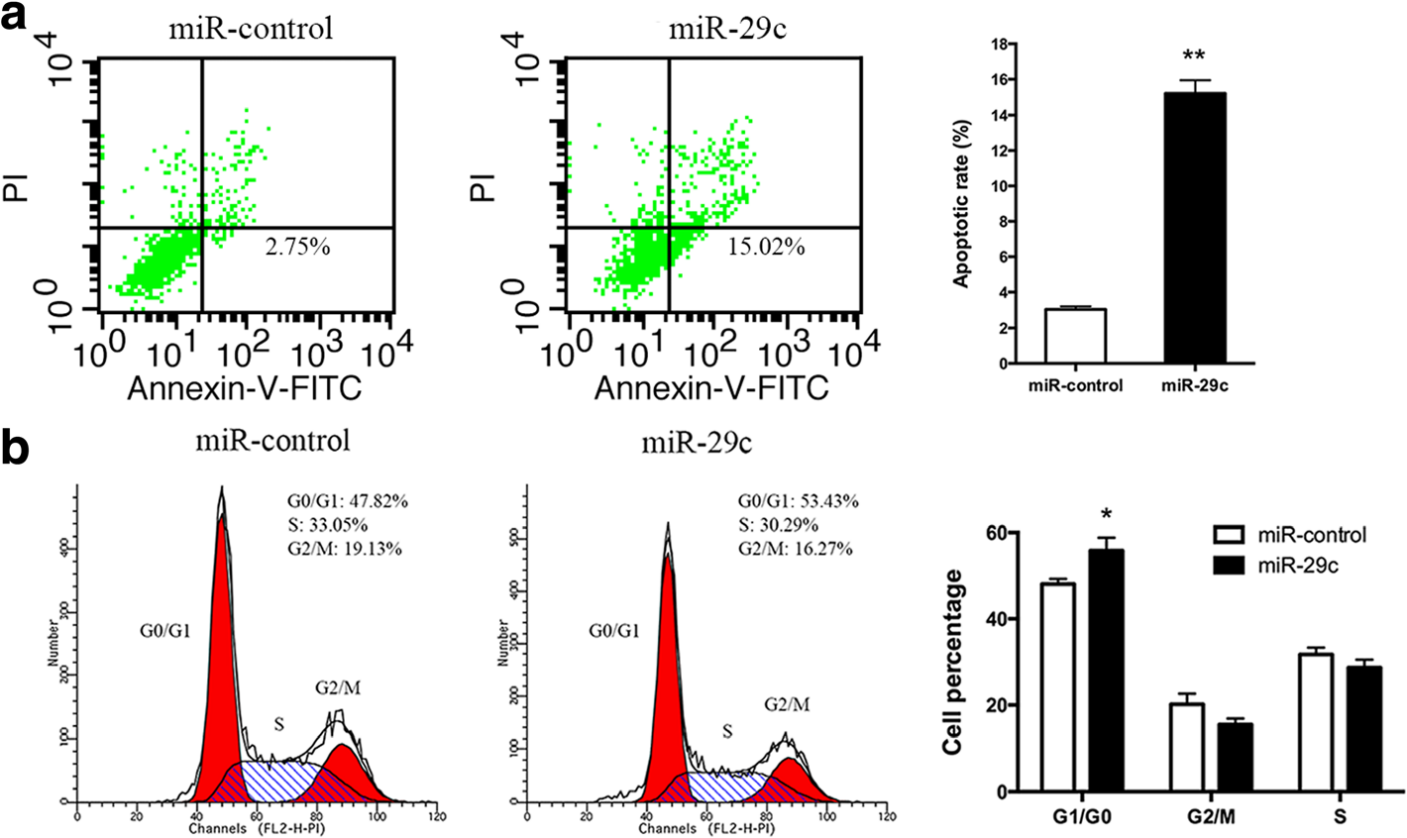
The effect of miR-29c on SGC-7901 apoptosis and cell cycle progression. **a** Representative histograms depicting apoptosis in SGC-7901 cells transfected with miR-29c or miR-control. Cells staining positive for Annexin V-FITC and negative for PI at 48 h after transfection were considered to have undergone apoptosis. **b** Representative histograms depicting cell cycle profiles of SGC-7901 cells transfected with miR-29c or miR-control. The results are shown as mean ± SD from three independent experiments. * *P* < 0.05, ** *P* < 0.01

Next, the effects of miR-29c on cell apoptosis and cell cycle were examined through flow cytometry. Our data showed that the apoptotic rate was significantly increased in cells transfected with miR-29c mimics in comparison with miR-control (15.2% ± 1.29% vs. 3.02% ± 0.297%, *P* = 0.0025, Fig. 3a). Cell cycle analysis showed the percentage of cells in G1/G0 phase was increased from 48.1% ± 1.20% to 55.9% ± 2.99% (*P* = 0.032, Fig. 3b). The percentage of cells in S phase and G2/M phase appeared to reduce in miR-29c overexpression cells, however, these differences had no statistically significance (Fig. 3b). The results indicate that overexpression of miR-29c can induce gastric cancer cell apoptosis and cell cycle arrest in G1/G0 phase, which contributes to growth inhibitory properties of miR-29c.

To confirm the biological function of miR-29c, we also transfected SNU-1 cells with miR-29c inhibitor (anti-miR-29c). In contrast, inhibition of miR-29c in SNU-1 promoted cell proliferation, reduced cell apoptosis and decreased cell percentage in G1/G0 phase (Additional file 3: Figure S2).

### miR-29c targets NASP directly

**Fig. 4:**
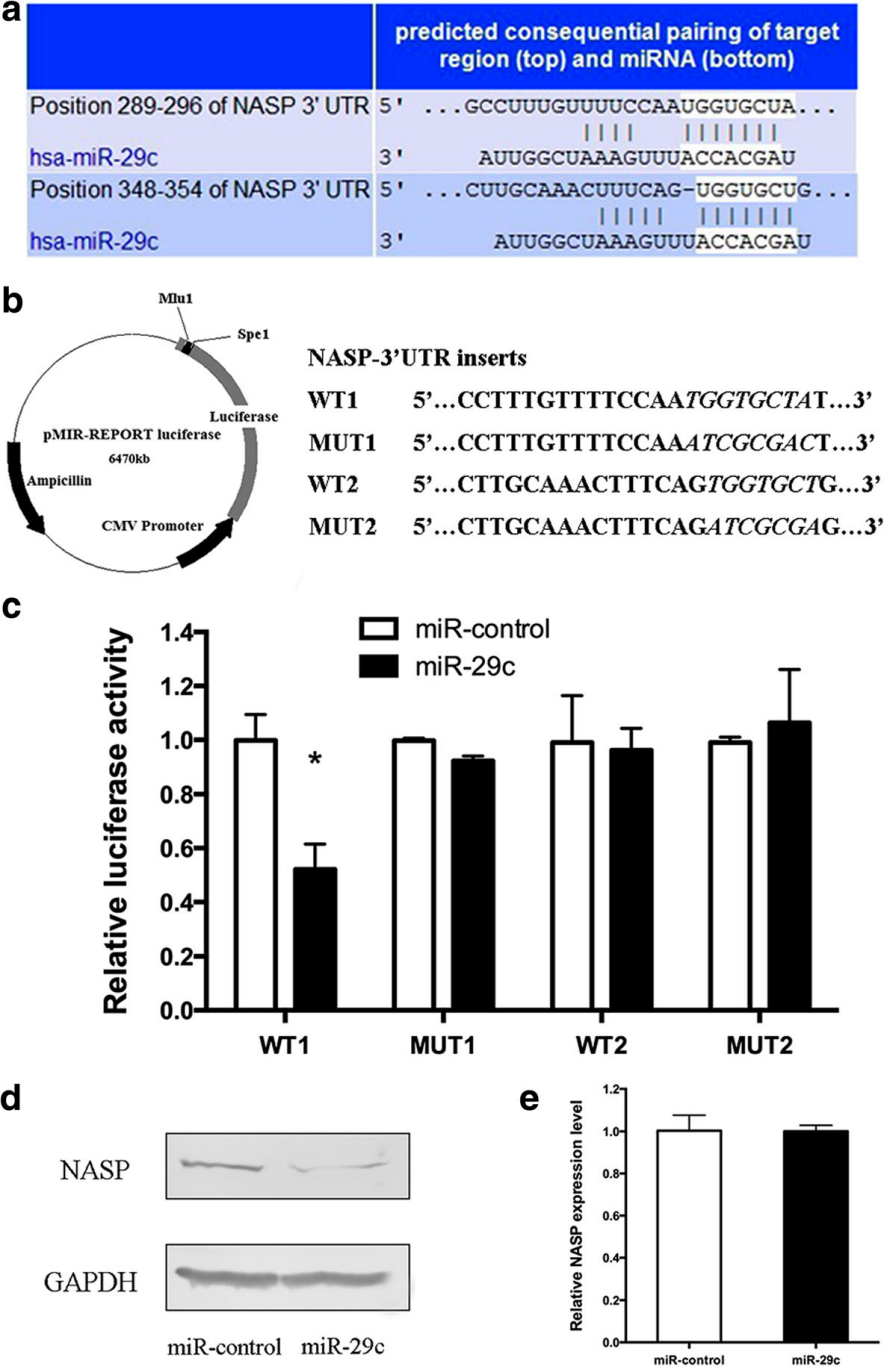
miR-29c targeted the 3′UTR of NASP and the effect on NASP expression. **a** Schematic graph of the putative binding sites of miR-29c in the NASP 3′UTR predicted by TargetScan. **b** Construction of the reporter plasmids. WT1, MUT1, WT2 and MUT2 were inserted into downstream of luciferase of pMIR-reporter vector. **c** Dual luciferase report assays were performed on HEK 293 T cells. **d**, **e** Forty-eight hours after miR-29c mimics or miR-control transfection on SGC-7901, NASP protein level and mRNA level was measured by Western blot analysis and qPCR, respectively. Each bar represents mean ± SD from three independent experiments. * *P* < 0.05

To explore the target gene of miR-29c by which inhibits cell proliferation in gastric cancer. We searched candidate target genes of miR-29c through TargetScan, miRBase Target and PicTar algorithms. Among the predicted targets, NASP, required for DNA replication, cell proliferation and normal cell cycle progression [11], was chosen as one of the targets of miR-29c and further identified two potential binding sites within its 3′UTR which located at position 289–296 (8-mer) and position 348–354 (7-mer), respectively (Fig. 4a).

To confirm the direct binding effect of miR-29c to NASP 3′UTR, dual luciferase report assay was performed in HEK 293 T. Two regions of NASP 3′UTR each containing one putative miR-29c binding site (named as WT1 and WT2) and their corresponding mutation types (named as MUT1 and MUT2) were cloned into the pMIR-Report luciferase vector (Fig. 4b). These constructs were cotransfected with miRNA mimics and pRL-TK into HEK 293 T cells, and luciferase activities were measured. For WT1 reporter transfected with miR-29c mimics, the luciferase activity was significantly decreased (*P* < 0.05), while for WT2 reporter, the activity was not affected after miR-29c transfection. Moreover, the repression of luciferase activity caused by miR-29c overexpression was clearly abrogated by MUT1 (Fig. 4c). These data indicate that miR-29c may target NASP through the 8-mer seeding region of 3′UTR.

Furthermore, we checked the influence of miR-29c on the expression of NASP by dectecting the protein and mRNA level of NASP after transfecting miR-29c mimics or miR-control. Western blot analysis displayed that an enforced miR-29c expression resulted in a decrease of NASP protein level in SGC-7901 cells (Fig. 4d). However, there was no effect on NASP mRNA level as detected by qPCR (Fig. 4e). These data demonstrate that miR-29c represses NASP expression at the post-transcriptional level, likely through directly targeting the 3′UTR of NASP.

### miR-29c suppresses tumorigenicity in vivo

**Fig. 5:**
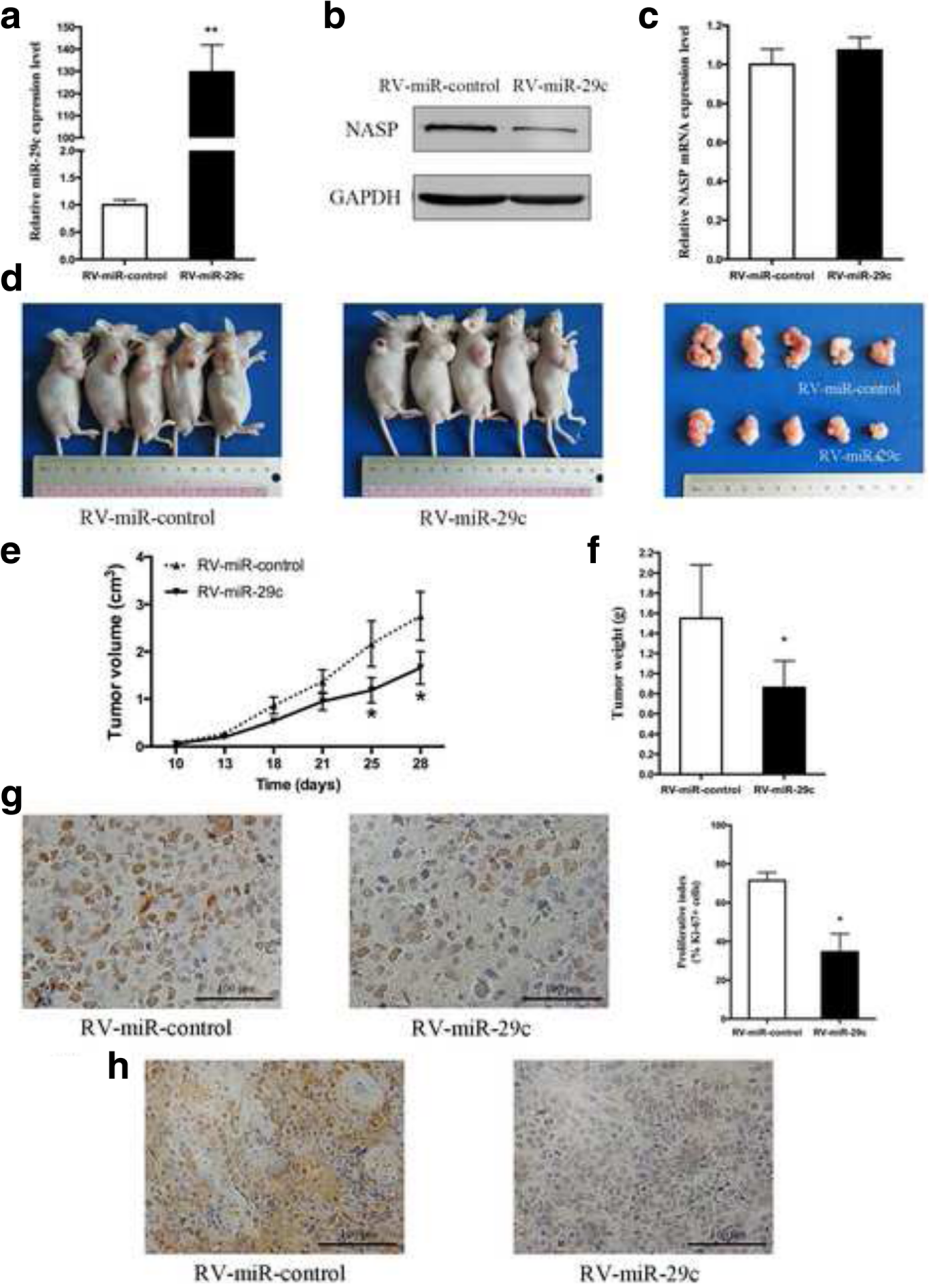
miR-29c inhibited tumorigenicity and proliferation in vivo. **a** qPCR analysis of miR-29c expression levels in SGC-7901-RV-miR-control and SGC-7901-RV-miR-29c cells. **b** Western blot analysis of NASP in SGC-7901-RV-miR-control and SGC-7901-RV-miR-29c cells. **c** qPCR analysis of NASP mRNA levels in SGC-7901-RV-miR-control and SGC-7901-RV-miR-29c cells. **d** Photographs of tumors derived from nude mice injected with SGC-7901-RV-miR-control and SGC-7901-RV-miR-29c cells. **e** Tumor growth kinetics in two groups of nude mice. **f** Average weight of tumor derived from nude mice injected with SGC-7901-RV-miR-control and SGC-7901-RV-miR-29c cells. **g** Representive photographs of immunohistochemical analysis of Ki-67 and its proliferation index in tumor xenografts. **h** Representive photographs of immunohistochemical analysis of NASP in tumor xenografts. Data are shown as mean ± SD of three independent experiments. * *P* < 0.05, ** *P* < 0.01

Next, we examined whether miR-29c overexpression could suppress the tumor growth in vivo. SGC-7901 cells mediated with RV-miR-29c or SGC-7901-RV-miR-control retrovirus were obtained as described in the Material and methods. The percentage of GFP positive cells was over 90% in both cell lines (Additional file 2: Figure S1B). And the miR-29c expression level was 129.7 ± 12.24 fold higher in SGC-7901-RV-miR-29c cells than that in control cells (*P* = 0.003, Fig. 5a). The result from Western blot was confirmed that the protein level of NASP was indeed down-regulated in SGC-7901-RV-miR-29c cells (Fig. 5b), and the NASP mRNA level still had no change after RV-miR-29c infection (Fig. 5c). These two groups of cells were injected subcutaneously into the right flank of nude mice and tumor nodules were monitored. After 4 weeks, the mice were sacrificed and the tumor nodules were weighted. As shown in Fig. 5d, tumors grew slower in the SGC-7901-RV-miR-29c group than those in the control group. The average tumor volume in SGC-7901-RV-miR-29c group at day 25 and day 28 was significantly smaller compared with the miR-control group (Fig. 5e). Similarly, the tumor weight in SGC-7901-RV-miR-29c group was significantly less than that from the miR-control group (1.550 ± 0.530 g vs. 0.860 ± 0.265 g, *P* = 0.041, Fig. 5f).

To assess whether tumor growth inhibition in mice injected with SGC-7901-RV-miR-29c cells was partly caused by the suppression of proliferation, IHC analysis of Ki-67 antigen was performed. As Fig. 5g shown, the percentage of Ki-67 positive cells was much lower in the nodules derived from SGC-7901-RV-miR-29c group than that in the control group (34.7% ± 9.29% vs. 71.3% ± 4.16%, *P* = 0.011). So the reduced tumor growth in mice was, at least in part, because of decreased proliferation which caused by miR-29c overexpression. Moreover, the tumor derived from RV-miR-29c showed weeker immunohistochemical staining of NASP than that derived from the control group (Fig. 5h). Therefore, miR-29c can inhibit tumorigenicity in vivo.

### miR-29c expression negatively correlates with NASP protein expression in gastric cancer cell lines

**Fig. 6:**
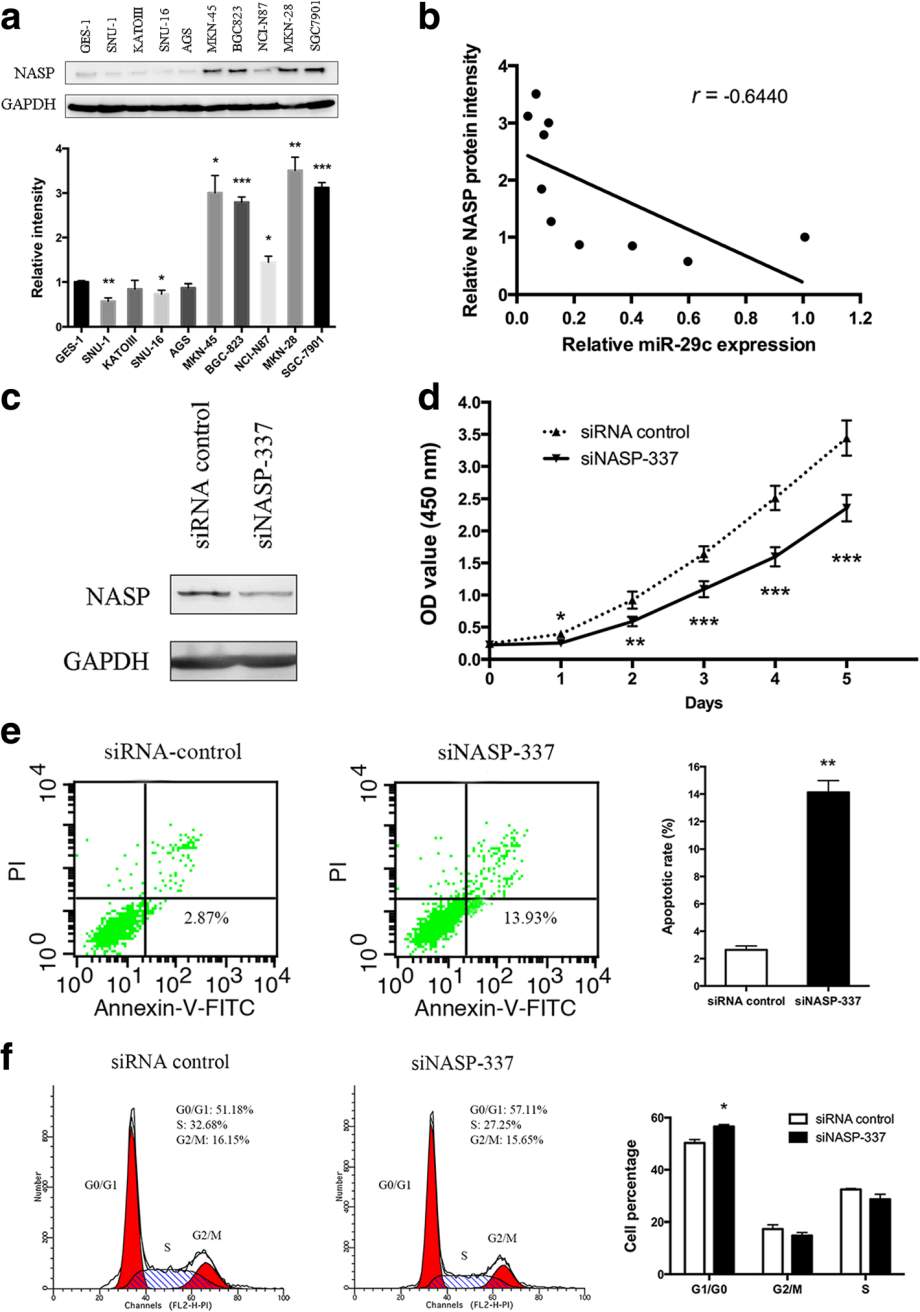
NASP knockdown elicits the phenotypes of miR-29c in gastric cancer cells. **a** NASP protein levels in GES-1 and 9 gastric cancer cell lines. The relative intensity was normalized to GES-1 and determined by Image J. **b** Scatter plot of miR-29c expression versus NASP protein expression in GES-1 and 9 gastric cancer cell lines. **c** Western blot analysis in SGC-7901 cells transfected with siNASP-337 or siRNA control to evaluate NASP knockdown efficiency. **d** CCK-8 proliferation assay was performed in SGC-7901 cells transfected with siNASP-337 or siRNA control. **e** Apoptosis assay in SGC-7901 cells transfected with siNASP-337 or siRNA control by flow cytometry. **f** Cell cycle progression was assessed by flow cytometry in SGC-7901 cells transfected with siNASP-337 or siRNA control. Data are represents as mean ± SD from three independent experiments. * *P* < 0.05, ** *P* < 0.01, *** *P* < 0.001

Next, we evaluated NASP protein level in GES-1 and nine gastric cancer cell lines by Western blot analysis. Most of the gastric cancer cell lines have an increase in the levels of NASP protein level compared with GES-1 (Fig. 6a). Among them, SGC-7901, MKN-45, MKN-28 and BGC823 expressed much higher levels of NASP while these cell lines exhibited lower miR-29c expression levels (Fig. 1c). Furthermore, there was a negative correlation between miR-29c and NASP protein level (*r* = −0.644, *P* = 0.044, Fig. 6b). Moreover, we measured miR-29c expression and NASP protein level in 4 pairs of gastric cancer and matched normal tissues. An inverse correlation was observed between miR-29c and NASP protein expression level, as shown in Additional file 2: Figure S1C.

### Knockdown of NASP elicits the phenotypes caused by miR-29c overexpression in gastric cancer cells

Since miR-29c down-regulated NASP to suppress cell proliferation, induce cell apoptosis and cause cell cycle arrest in G1/G0, it is reasoned that specific knockdown of NASP could elicit similar phenotypes induced by miR-29c in gastric cancer. To test it, SGC-7901 were transfected with NASP siRNA firstly, and the knockdown efficiency was confirmed by qPCR and Western blot (Additional file 2: Figure S1D). Among three siRNAs of NASP, siNASP-337 was chose for further analysis because of its highest knockdown efficiency (Fig. 6c). As expected, proliferation assay showed that knockdown of NASP have an obvious inhibitory effect on SGC-7901 cell growth (Fig. 6d). Cell apoptosis rate raised from 2.64% ± 0.502% to 14.1% ± 1.50% after transfection with siNASP-337 (*P* = 0.003, Fig. 6e). Moreover, cell cycle progression was also arrested in G1/G0 phase (56.6% ± 0.785% vs. 50.2% ± 1.29%, *P* = 0.042, Fig. 6f). Further, NASP was overexpressed in SGC-7901 cells and then transfected with miR-29c mimics, the inhibitory effect of miR-29c on cell proliferation was partially reversed, and the progressions toward apoptosis and G1/G0 cell cycle arrest were also hindered (Additional file 4: Figure S3). Taken together, these data provide evidence that the function of miR-29c inhibit cell proliferation in gastric cancer is at least partially through targeting NASP.

## Discussion

Accumulating studies have focused on the role of miRNAs play in regulating cell proliferation process in gastric cancer. MiR-29c has been shown to be down-regulated in gastric cancer, but the downstream targets differ and it is not clear how miR-29c mediates cell responses in varying cell contexts. Han et al. reported that miR-29c involved in the initiation of gastric carcinogenesis by directly targeting ITGB1 [12]. They found restoration of miR-29c inhibits cell proliferation, adhesion, invasion and migration in gastric cancer. Another research group verified the downregulation of miR-29c in gastric cancer patients and assessed proliferation and colony formation ability of miR-29c by targeting RCC2 [13]. It was showed that all the members of miR-29 family were down-regulated in gastric cancer and miR-29c was more significant as a signature miRNA than miR-29a or 29b for gastric cancer. Furthermore, they demonstrated that miR-29 family directly targeted CCND2 and MMP2 to influence gastric cancer progression [14]. It also has been reported that miR-29c regulates the expression of many oncogenes, such as CDK6, CDC42, p85α, DNMT3a and DNMT3b in other types of cancers [6, 15, 16].

In present study, we indicate that miR-29c acts as a tumor suppressor by suppressing cell growth through CCK-8 and colony formation assays, promotes apoptosis and arrests cell cycle at G1/G0 stage by targeting NASP. Furthermore, the down-regulation of NASP can elite the phenotypes caused by miR-29c. As a histone chaperone, NASP binds both core and linker histones that is proved to present in all dividing cells [17]. Two splice variants of NASP have been reported: testicular NASP (tNASP), which is mainly expressed in testis, stem cells, embryonic tissues and malignant tumors; somatic NASP (sNASP), which exists in all somatic mitosis cells. Both types of NASP specifically bind to histone H1, H3 and H4 and affect chromatin assembly, lead to the association with DNA replication, cell proliferation and cell cycle progression [18]. Previous studies have investigated the role of NASP in renal cell carcinoma [19]. Fang et al. showed that tNASP has a relative high level in human renal cell carcinoma and tNASP knockdown effectively suppresses cell proliferation and induces G1 phase arrest through ERK/MAPK signaling pathway. Additional studies indicated that depletion of tNASP inhibited cell proliferation and promoted apoptosis in prostate cancer cells [20]. However, the mechanisms that result in elevated NASP expression level are still unclear. Our study suggests one mechanism that contributes to the elevated NASP levels in tumors is a deregulation of miR-29c and further supports targeting NASP as a therapeutic strategy in gastric cancer.

Our study also demonstrates that the expression level of miR-29c is lower in 67 cases of gastric cancer compared with matched normal tissues, and the expression also decreases in nine gastric cancer cell lines versus GES-1. The relationship between the miR-29c expression level and the clinicopathological factors in human gastric cancer samples was further analyzed. However, miR-29c expression level did not show any correlation with the clinicopathological parameters. It is consistent with the result of our previous study [21]. Among 15 candidate miRNAs selected from microRNA array, miR-29c showed no correlation with the clinicopathological features assessed by qPCR in 40 pairs of gastric cancer samples. Liu et al. evaluated the role of miR-29c, miR-124, miR-135a and miR-148a in predicting lymph node metastasis and tumor stage in gastric cancer, they showed a week relationship between miR-29c expression level and gastric cancer stage on the basis of *P* = 0.049 in 60 gastric cancer tissues [22]. More samples and further studies are needed to disclosure the relationship between miR-29c and the clinicopathological features in gastric cancer.

## Conclusions

In summary, we have clarified a novel pathway regulating cell proliferation in gastric cancer, which is, miR-29c inhibits cell proliferation, promotes apoptosis and arrests cell cycle at G1/G0 phase by targeting NASP. Our study highlights the potential apply of miR-29c as an early biomarker and therapeutic target of gastric cancer.

## Additional files

Additional file 1: Table S1. Relationship between miR-29c expression level and clinicopathologic features in 67 gastric cancer tissues. (DOC 41 kb)
Additional file 2: Figure S1. (A) Transfection efficiency was monitored by using a Cy3-labeled pre-miR negative control. (B) GFP positive cells in SGC-7901-RV-miR-control and SGC-7901-RV-miR-29c cells detected by flow cytometry. (C) QPCR and western blot analysis of NASP in four paired tumor/normal tissues (T/N). (D) NASP knockdown efficiency was evaluated by qPCR and Western blot. (TIF 1493 kb)
Additional file 3: Figure S2. Inhibition of miR-29c by anti-miR-29c in SNU-1 cells down-regulated NASP expression (A), promoted cell proliferation (B), reduced apoptosis (C) and decreased cell percentage at G1/G0 phase (D). (TIF 506 kb)
Additional file 4: Figure S3. NASP expression vector was transfected into SGC-7901 cells (A). Overexpression of NASP rescued the effect of miR-29c in gastric cancer cells, including cell growth (B), cell apoptosis (C) and cell cycle analysis (D). (TIF 698 kb)

## Abbreviations

3′UTR: 3′untranslated regions
miR-29c: microRNA-29c
miRNAs: microRNAs
NASP: Nuclear autoantigenic sperm protein
sNASP: somatic NASP
tNASP: testicular NASP
WST: Water-soluble tetrazolium
