# Supplementary material for: microRNA-29c inhibits cell proliferation by targeting NASP in human gastric cancer
Source: BMC Cancer. 2017 Feb 7;17:109. doi: 10.1186/s12885-017-3096-9 (PMC5294820; doi:10.1186/s12885-017-3096-9)
Supplement: Additional file 1: Table S1. — Relationship between miR-29c expression level and clinicopathologic features in 67 gastric cancer tissues. (DOC 41 kb) [file 12885_2017_3096_MOESM1_ESM.doc]

**Table S1** Relationship between miR-29c expression level and clinicopathologic features in 67 gastric cancer tissues

| **Clinicopathologic parameters** | **miR-29c expression** | | ***P*-value** |
| --- | --- | --- | --- |
|  | **Low (n = 32)** | **High (n = 35)** |  |
| Gender |  |  |  |
| Male | 19 | 20 | 0.8532 |
| Female | 13 | 15 |  |
| Age (years) |  |  |  |
| ≤ 60 | 19 | 18 | 0.5135 |
| > 60 | 13 | 17 |  |
| Location |  |  |  |
| Distal third | 22 | 24 | 0.9874 |
| Middle third, proximal third | 10 | 11 |  |
| Borrmann classification |  |  |  |
| I, II | 13 | 15 | 0.8532 |
| III, IV | 19 | 20 |  |
| Differentiation |  |  |  |
| Poorly, undifferentiated | 22 | 28 | 0.2905 |
| Well, moderatelly | 10 | 7 |  |
| Local invasion |  |  |  |
| T1, T2 | 5 | 5 | 0.8779 |
| T3, T4 | 27 | 30 |  |
| Lymph node metastasis |  |  |  |
| No | 7 | 8 | 0.9233 |
| Yes | 25 | 27 |  |
| TNM stage |  |  |  |
| I, II | 10 | 11 | 0.9874 |
| III, IV | 22 | 24 |  |
