# Supplementary figures and images for: microRNA-29c inhibits cell proliferation by targeting NASP in human gastric cancer
Source: BMC Cancer. 2017 Feb 7;17:109. doi: 10.1186/s12885-017-3096-9 (PMC5294820; doi:10.1186/s12885-017-3096-9)

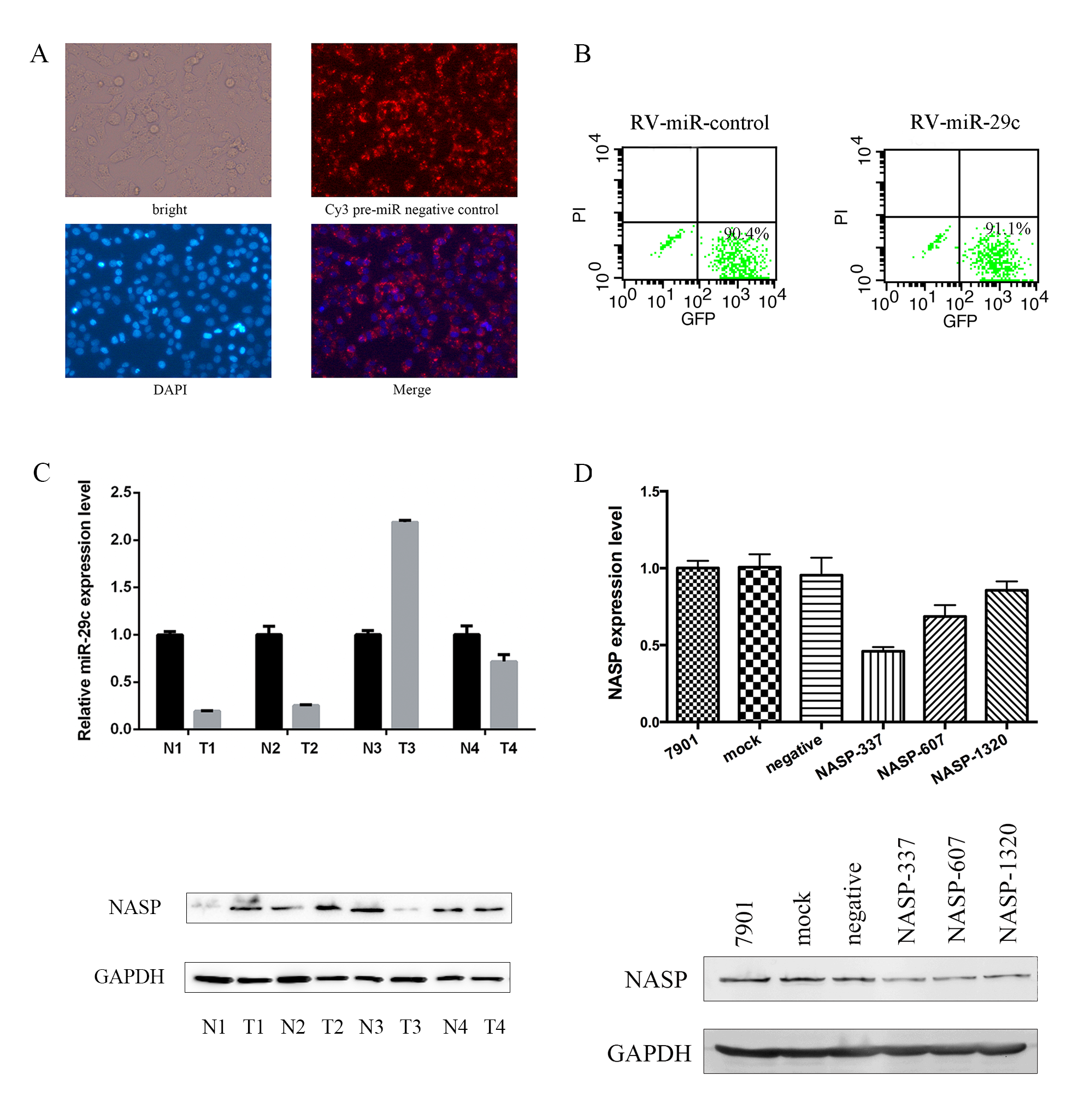

Supplement: Additional file 2: Figure S1. — (A) Transfection efficiency was monitored by using a Cy3-labeled pre-miR negative control. (B) GFP positive cells in SGC-7901-RV-miR-control and SGC-7901-RV-miR-29c cells detected by flow cytometry. (C) QPCR and western blot analysis of NASP in four paired tumor/normal tissues (T/N). (D) NASP knockdown efficiency was evaluated by qPCR and Western blot. (TIF 1493 kb) [file 12885_2017_3096_MOESM2_ESM.tif]

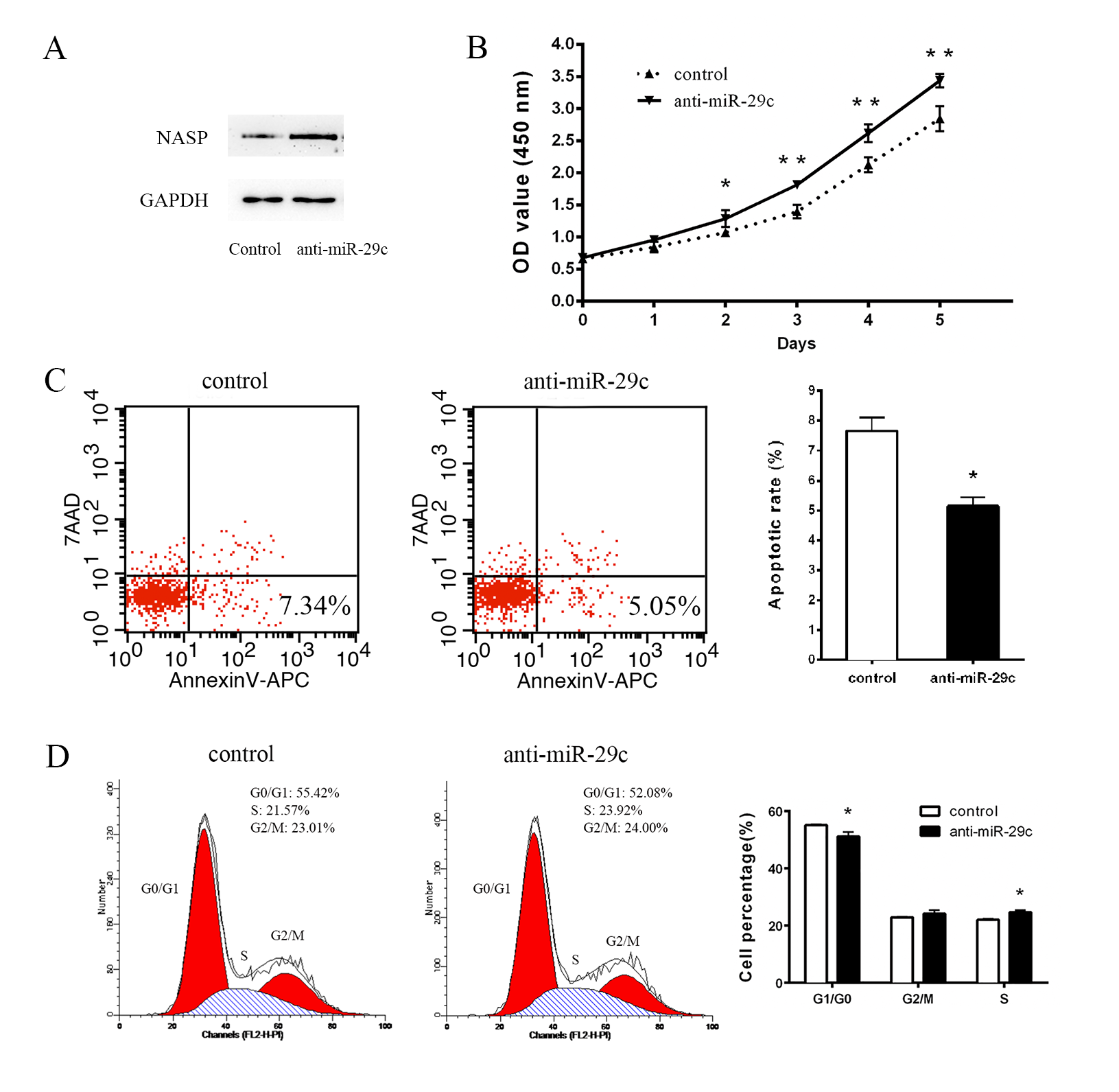

Supplement: Additional file 3: Figure S2. — Inhibition of miR-29c by anti-miR-29c in SNU-1 cells down-regulated NASP expression (A), promoted cell proliferation (B), reduced apoptosis (C) and decreased cell percentage at G1/G0 phase (D). (TIF 506 kb) [file 12885_2017_3096_MOESM3_ESM.tif]

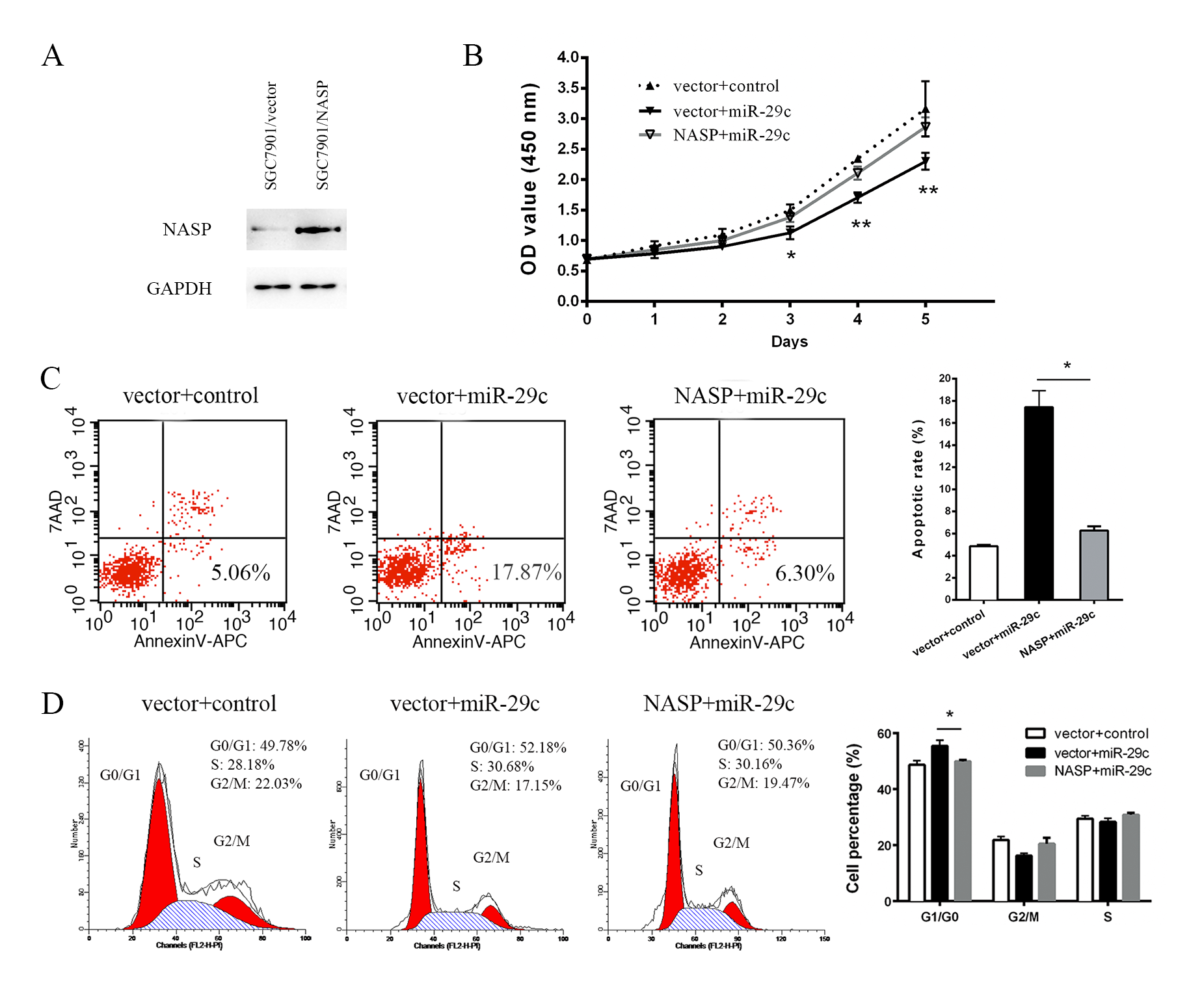

Supplement: Additional file 4: Figure S3. — NASP expression vector was transfected into SGC-7901 cells (A). Overexpression of NASP rescued the effect of miR-29c in gastric cancer cells, including cell growth (B), cell apoptosis (C) and cell cycle analysis (D). (TIF 698 kb) [file 12885_2017_3096_MOESM4_ESM.tif]
